# Effect of a pro-breastfeeding intervention on the maintenance of breastfeeding for 2 years or more: randomized clinical trial with adolescent mothers and grandmothers

**DOI:** 10.1186/s12884-016-0878-z

**Published:** 2016-05-03

**Authors:** Cristiano Francisco da Silva, Leandro Meirelles Nunes, Renata Schwartz, Elsa Regina Justo Giugliani

**Affiliations:** Graduate Program in Child and Adolescent Health, School of Medicine, Universidade Federal do Rio Grande do Sul, Porto Alegre, RS Brazil; CLN 115 Bloco C Apt 117 m, CEP 70772-530 Brasília, DF Brazil

**Keywords:** Breastfeeding, Controlled randomized clinical trial, Adolescent, Infant nutrition

## Abstract

**Background:**

Being an adolescent mother and cohabiting with the maternal grandmother have been shown to be risk factors for a shorter breastfeeding duration. The objective of this study was to assess whether the positive effects of a pro-breastfeeding intervention aimed at adolescent mothers and maternal grandmothers on the prevalence of breastfeeding observed in the first year of life were maintained at 2 years of age.

**Methods:**

This study is the continuation of a randomized clinical trial initiated in 2006 involving 323 adolescent mothers, their newborns and maternal grandmothers when cohabiting. The intervention consisted of six breastfeeding counseling sessions, the first one held at the maternity ward and the others at the participants’ homes at 7, 15, 30, 60, and 120 days postpartum. The present study reports data collected when the children were 4 to 7 years old, concerning the maintenance of breastfeeding at 2 years. Data were analyzed using multivariable Poisson regression model with robust variance, with breastfeeding at 2 years of age as the outcome.

**Results:**

Maintenance of breastfeeding for 2 years or more was present in 32.2 % of the sample. When the intervention and control groups were compared, the prevalence of breastfeeding at 2 years was similar (29.9 vs. 34.3 %, respectively; *p* = 0.605). Multivariable analysis failed to reveal an association between exposure to the intervention and maintenance of breastfeeding at 2 years in the different models tested.

**Conclusions:**

The positive impact of the intervention on the prevalence of breastfeeding observed in the first year of life was not maintained at 2 years of age.

**Trial registration:**

The study was registered at ClinicalTrials.gov on May 28, 2009 under protocol no. NCT00910377.

## Background

The benefits of breastfeeding have been consistently demonstrated, both for the children and for the women who breastfeed [1–4]. Several of these benefits have been suggested to be dose-dependent, i.e., the greater the exposure to breastfeeding, the greater the benefits. Examples of dose-dependent benefits associated with breastfeeding include a lower chance of developing overweight/obesity [5] and improved cognitive development [6] in individuals who were breastfed for longer periods, as well as a lower risk of breast cancer [7] and type 2 diabetes [8] in women who have breastfed. Nevertheless, the number of women who comply with the recommendation of the World Health Organization (WHO) to breastfeed for 2 years or more is still low [9]. In Brazil, half of the women maintain breastfeeding up to 12 months, and only one fourth until 2 years [10].

Taking into consideration the need to test strategies aimed to increase the duration of breastfeeding and exclusive breastfeeding in Brazil, a randomized clinical trial was conducted in the city of Porto Alegre, southern Brazil, with the objective of assessing the effectiveness of a pro-breastfeeding intervention aimed at adolescent mothers and maternal grandmothers. The decision to involve adolescents and maternal grandmothers was based on the results of previous studies indicating that being an adolescent mother and cohabiting with the maternal grandmother are risk factors for a shorter breastfeeding duration [11–13].

The intervention has already proved effective in increasing the duration of exclusive breastfeeding [14] and the prevalence of breastfeeding in the first year of life [15], in delaying and reducing the unnecessary intake of water and/or herbal teas by breastfed infants [16], and has also had a positive impact against the early introduction of complementary foods [17]. The objective of the present study was to assess whether the positive effects of the intervention observed in the first year of life in terms of the prevalence of breastfeeding were maintained at 2 years of age.

## Methods

This study is the continuation of a randomized clinical trial initiated in 2006 involving 323 adolescent mothers, their newborns and also their mothers (child’s maternal grandmothers) whenever cohabiting with the former. Sample selection procedures and intervention details are described elsewhere [15]. Briefly, adolescent mothers were selected at the inpatient obstetric ward of Hospital de Clínicas de Porto Alegre (HCPA) observing the following inclusion criteria: being 19 years old or younger, residing in the city of Porto Alegre, and breastfeeding their babies. Mothers of newborns weighing less than 2,500 g, of twins, or of newborns with congenital defects that could interfere with breastfeeding were excluded, as were mothers living with their mothers-in-law (paternal grandmothers).

Since the original clinical trial was planned to evaluate another question (rates of exclusive breastfeeding and breastfeeding in the first year of life), we calculated the effect size that can be detected with the sample available at the follow up assessment (*n* = 207), considering the new question. Thus, estimating a prevalence of 32 % of breastfeeding at 2 years in the group not exposed to the intervention [18], this sample size was considered large enough to detect a difference of 20 percentage points or higher in the prevalence of breastfeeding between the group exposed and the one not exposed to the intervention, with alpha error at 5 % and beta error at 20 %.

Following subject selection, adolescents were allocated to either the control or the intervention group by block random allocation in groups of two (block size 2). Further randomization details are described elsewhere [15].

The intervention consisted of six breastfeeding counseling sessions, all held by the same person, one out of four health professionals trained to do so, namely, a pediatrician, two nurses, and a nutritionist, all with extensive knowledge and expertise in breastfeeding counseling (three of them were certified international lactation consultants). The first counseling session lasted for approximately 1 h and was held at the maternity ward, individually, at different times for the mothers and grandmothers. The intervention with the grandmothers followed the same principles adopted for the mothers, however covering additional topics that underscored the role of grandmothers for a successful breastfeeding. Subsequent sessions were held at the participants’ homes at 7, 15, 30, 60, and 120 days postpartum, in the presence of both the mother and the grandmother, when cohabiting. The WHO principles of breastfeeding counseling were followed [19], namely, establishing a dialog between mothers, grandmothers, and health professionals on different aspects of breastfeeding, e.g., its importance; frequency and duration of feeds; recommended duration (6 months of exclusive breastfeeding and 2 years or more of any breastfeeding); factors that interfere with milk supply; breastfeeding techniques; consequences of dummy use and bottle-feeding; infant crying and communication; and specific doubts expressed by the mothers and/or grandmothers. During the sessions held at the maternity ward, mothers were stimulated to breastfeed whenever possible, so as to create an opportunity for the interviewer to observe the feed and offer guidance on adequate positioning and latch, when appropriate. At the households, the sessions focused on the difficulties faced by the mothers with infant feeding and breastfeeding management, and key messages addressed in the first session (at the maternity ward) were reinforced. Flip charts were created, one for the mother and one for the grandmother, and a booklet covering several aspects of breastfeeding (including the recommended duration of 2 years or more) was given to the mothers in the first session, at the maternity ward.

Data were collected at different time points. At the maternity ward, once the adolescent mothers and the maternal grandmothers agreed to participate in the study, they were interviewed individually by the same professionals responsible for the intervention to collect data on sociodemographic characteristics and aspects related to prenatal care, delivery, and previous experience with breastfeeding. Different questionnaires were used for the mothers and for the grandmothers. Data on infant feeding in the first year of life were obtained monthly in the first 6 months, every 2 months between 6 and 12 months of age, always via telephone contact or home visit (whenever telephone contact failed). These data were collected by research assistants who were blind to group allocation. In order to assess the quality of data collection, 5 % of the mothers were drawn and subjected to a second interview with the lead field researcher, containing some key questions of the follow-up questionnaire.

When the children were 4 to 7 years old, the mother-child dyads were contacted again for a new assessment. Contact with the research participants was attempted via telephone contact, review of medical records from the hospitals where the children were born, search in online social networks, and ultimately home visits. Once the mothers were located, they were invited to visit the clinic, bringing along their children, on a given date. Whenever the mother reported not being able or failed to attend the appointment, the families were visited at their homes. At this occasion, the mothers were interviewed once again, and information was obtained on the duration of breastfeeding and infant feeding practices. Updated data on the mother, the child, and the family were also collected.

The characteristics of the children lost to follow-up were compared with those of the children who remained in the study using inferential analysis, as were the characteristics of the control and intervention groups. Means and proportions were compared using Student’s *t* or Mann-Whitney’s test and Pearson’s chi-square or Fisher’s exact test, respectively. Multivariable Poisson regression model with robust variance was used with a model that included variables showing differences between the control and intervention groups as a result of the losses (*p* < 0.20). After the use of a non-adjusted model, different cumulative models were tested, progressively including variables of the previous model(s). The first adjusted model considered only group and propensity score (Seeger et al. [20]). The propensity scores were estimated using logistic regression, modeling the probability of an individual being allocated to the intervention group and considering the following predictors: maternal age, educational attainment, skin color, and parity; infant weight and mode of delivery; and parental cohabitation. Significance was set at 5 % (*p* ≤ 0.05). All analyses were performed using the Statistical Package for the Social Sciences (SPSS), version 21.0.

All mothers and grandmothers were informed of the study objectives and were included only after signing a written informed consent form, both at baseline and at the latest evaluation. Whenever the adolescent mother was younger than 18 years, both she and a parent/guardian signed the consent form. The anonymity of participants and the use of results for research purposes only was guaranteed. The study was approved by the Research Ethics Committee of Hospital de Clínicas de Porto Alegre, and was registered at ClinicalTrials.gov under protocol no. NCT00910377.

## Results

**Fig. 1:**
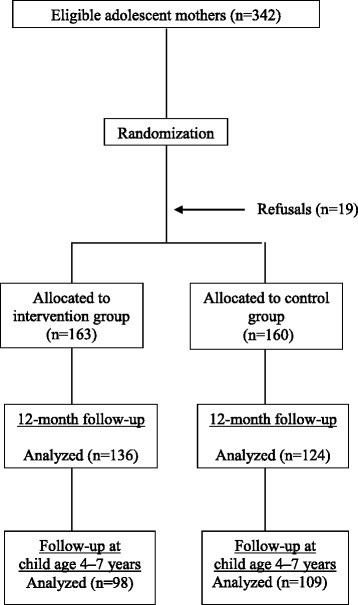
Flow chart of the randomized clinical trial phases from sample selection to the latest evaluation

Figure 1 shows all the phases of the randomized clinical trial from sample selection to the latest assessment (data shown here), when children were aged 4 to 7 years. Of the 323 mothers included at the start of the trial, 207 (64.1 %) were located at the present stage, namely, 98 (46.9 %) from the intervention group and 109 (53.1 %) from the control group.

Table 1 presents the characteristics of the population at the time of the intervention and at the latest evaluation, stratified by group allocation. Even though this was a randomized clinical trial, the number of mothers lost to follow-up and changes in some of the sociodemographic characteristics assessed resulted in differences between the intervention and control groups, namely, cohabitation with maternal grandmother at the beginning of the study, and child’s age and mother’s education level at the latest assessment. The other characteristics were similar between the two groups.

**Table 1:** Characteristics of participants who completed the study stratified by group allocation

Variable	Intervention	Control	*p*
	(*n* = 98)	(*n* = 109)	
At the time of the intervention			
Maternal age (years) – mean ± SD	17.4 ± 1.5	17.5 ± 1.4	0.675
Maternal educational attainment ≥ 8 years – n (%)	55 (56.1)	55 (50.5)	0.499
Infant birth weight (g) – mean ± SD	3252 ± 421	3252 ± 428	0.995
Per capita income (MW^a^) – median (interquartile range)	0.5 (0.3–0.6)	0.4 (0.2–0.6)	0.685
Infant sex, male – n (%)	45 (45.9)	56 (51.4)	0.519
Maternal skin color, white – n (%)	62 (63.3)	67 (61.5)	0.902
Mode of delivery, vaginal – n (%)	73 (74.5)	81 (74.3)	1.000
Primiparity – n (%)	88 (89.8)	89 (81.7)	0.143
Cohabiting with partner – n (%)	57 (58.2)	68 (62.4)	0.633
Cohabiting with maternal grandmother – n (%)	64 (65.3)	53 (48.6)	0.023
At the latest assessment			
Maternal age (years) – mean ± SD	23.9 ± 4.0	24.4 ± 1.7	0.305
Child age (years) – mean ± SD	5.82 ± 0.52	6.30 ± 0.36	<0.001
Per capita income (MW) – median (interquartile range)	0.6 (0.4–0.9)	0.6 (0.4–0.8)	0.814
Other children born – n (%)	36 (36.7)	42 (38.9)	0.861
Maternal educational attainment, ≥ 8 years – n (%)	76 (80.9)	72 (67.3)	0.044
Maternal employment outside the home – n (%)	49 (52.1)	63 (58.3)	0.457
Cohabitation with maternal grandmother – n (%)	30 (31.3)	25 (23.4)	0.270
Cohabitation with paternal grandmother – n (%)	3 (3.2)	10 (9.3)	0.138
Cohabitation with partner – n (%)	57 (60.6)	76 (71.0)	0.160

*SD* Standard deviation

^a^MW = minimum wage (US$ 195.00 at the time of the study)

Approximately one third of the children (*n* = 66; 32.2 %) were breastfed for at least 2 years. When the intervention and control groups were compared, the prevalence of breastfeeding at 2 years was similar (29.9 vs. 34.3 %, respectively; *p* = 0.605).

Table 2 shows the results of the multivariable analysis used to assess the effect of the intervention on the maintenance of breastfeeding at 2 years of age. No significant effect was found on the likelihood of a child being breastfed at 2 years, even after control for possible confounders.

**Table 2:** Poisson regression with robust variance estimation to assess the effect of the intervention on maintenance of breastfeeding at 2 years of age

Model	RR (95 % CI)	*p*
1 – Intervention group	0.87 (0.58–1.30)	0.506
2 – Model 1 + propensity score^a^	0.93 (0.62–1.40)	0.722
3 – Model 2 + cohabitation with grandmother^b^	0.91 (0.60–1.37)	0.643
4 – Model 3 + child’s age^b^	0.90 (0.57–1.43)	0.668
5 – Model 4 + mother’s education level^b^	0.88 (0.55–1.40)	0.580

*RR* Relative risk, *95 % CI* 95 % confidence interval

^a^Variables covered by the propensity score: maternal age, education level, skin color, and parity; child’s birth weight and type of delivery; and cohabitation between child’s mother and father; all referring to the beginning of the study

^b^Upon the latest assessment

## Discussion

Over the past few years, there has been increasing evidence of the effectiveness of health interventions designed to increase the rates of breastfeeding and exclusive breastfeeding in the population via different support and promotion strategies aimed at lactating mothers [21]. However, the great majority of the interventions is targeted at adult women. Taking into consideration the peculiarities of adolescent motherhood, e.g. the need for the adolescents to shift from their role as daughters to the role of mothers, the instability of their love relationships, financial and emotional dependence on the family, higher rates of postpartum depression [1], as well as distortions of self-image and self-esteem typical of this age range [22], the results reported for adult women can rarely be extrapolated to adolescent mothers. In a recent systematic review conducted to assess the effect of pro-breastfeeding interventions on the rates of breastfeeding in adolescent mothers from developed countries, Sipsma et al. [23] pointed to the urgent need for more clinical trials designed to test new interventions targeted specifically at adolescent mothers. Moreover, those authors recommended the inclusion of the adolescent mothers’ mothers and partners in the interventions, a feature that was absent in the studies included in their review. In this sense, the present study has the strength of including, ever since its conception and design, adolescent mothers and maternal grandmothers when cohabiting.

Another strength of this study is the decision to use the breastfeeding duration recommended by the WHO, i.e., 2 years or more. To the authors’ knowledge, no previous intervention studies have investigated the maintenance of breastfeeding for 2 years or more, in part because of the low number of women who breastfeed their children for over 1 year, especially in developed countries, where most of the previous studies have been conducted.

As reported previously [15], the intervention here described was successful in increasing the prevalence of breastfeeding in the first year of life, especially when the adolescent mothers did not cohabit with the child’s maternal grandmothers. With the intervention, the chance of maintaining breastfeeding in the first year of life increased by 49 % in the group of adolescent mothers who did not live with grandmothers and by 26 % in the group of mothers who cohabited with grandmothers. These results raised the expectation that the intervention could also increase the prevalence of breastfeeding at 2 years of age. However, the hypothesis was not confirmed by the present findings. The prevalence of breastfeeding at 2 years of age was similar in both the intervention and the control groups, and, unlike the results found at 12 months, cohabitation with maternal grandmother did not have an influence at 2 years, as demonstrated by the multivariate analysis (Table 2).

One possible explanation for the absence of positive effects of the intervention at 2 years of age could be the long interval between the intervention and the outcome investigated. The last counseling session of the intervention was held when the child was 4 months old, followed by a 20 months’ interval with no intervention until the outcome. Moreover, the contents of the intervention may not have contemplated the peculiarities of determinants of breastfeeding maintenance for 2 years of more in adolescent mothers, which are unknown.

The fact that the intervention was successful in increasing the prevalence of breastfeeding in the first year of life but not at 2 years reinforces the hypothesis that some determinants of the maintenance of breastfeeding for 2 years or more differ from those of breastfeeding in the first year. The only previous study that has identified factors associated with breastfeeding for 2 years or more was conducted in the same setting as ours [18]. In that study, the following factors were associated with breastfeeding maintenance at 2 years: mother staying at home with the child in the first 6 months of life (RR = 2.13); not using a pacifier (RR = 2.45); later introduction of water and/or teas and of other milks in the child’s feeding; and mother not cohabiting with partner (RR = 1.39). It is interesting to note that, in that study, cohabitation with the maternal grandmother was not associated with maintenance of breastfeeding for 2 years or more, as also observed in the present study; in our sample, the relative risk of the impact of the intervention on the outcome did not significantly change when the variable cohabitation with maternal grandmother was introduced in the model. It is possible that those mothers who breastfeed for 2 years or more, including adolescent mothers, have more self-confidence and determination, and are therefore less susceptible to negative influence from people living with them.

Some limitations of this study should be discussed. First, there was an important number of mothers lost to follow-up, probably due to the long time of the follow-up period. Losses to follow-up are common in population-based studies involving youth living in the peripheries of developing countries. In order to minimize a possible selection bias due to these losses, the statistical model employed included variables showing different prevalence rates in the intervention and control groups.

Another possible limitation is the large age range (4 to 7 years) at follow up assessment. This happened because we did not determine a specific age for the follow up evaluation. Since we spent almost two years to recruit the sample and ten months to locate all families for the follow up assessment, we ended up having this wide age range. Nevertheless, we believe that this fact has not affected the results, especially as the child's age was considered in the multivariable analyses.

And finally, we can not disregard the possibility of recall bias, as data on the duration of breastfeeding were collected retrospectively for mothers breastfeeding for over 12 months (53.2 % of the sample). However, this type of bias is more relevant when investigating duration of exclusive breastfeeding [24], as mothers tend to recall the duration of breastfeeding with relative accuracy. According to a study conducted in the United States, breastfeeding duration was only slightly overestimated at 1 to 3.5 years after the outcome [25]. Moreover, in our study, the outcome did not focus on a specific date, but rather on a period (2 years or more), which probably reduces the potential negative impact of a recall bias.

## Conclusions

The positive impact of the intervention on the prevalence of breastfeeding observed in the first year of life was not maintained at 2 years of age. This result has raised new questions: What can we do to extend the positive impact of the intervention? Would it be enough to just increase exposure to the intervention? Would it be necessary to address issues specifically related to the maintenance of breastfeeding for 2 years of more in adolescent mothers, including the peculiarities of their life style? Would it be important to include the child’s father in the intervention, given previous findings showing that cohabitation with partner may inhibit maintenance of breastfeeding for 2 years of more [18]? Answers to these questions are important for the development of effective strategies at all levels of health care, with pro-breastfeeding policies aimed to follow the recommendation to breastfeed for 2 years or more, without disregarding that adolescent mothers require a different approach, one that addresses their peculiarities and particular needs. Promoting breastfeeding among adolescent mothers is particularly important in our setting, as a considerable portion of all births in Brazil are from adolescent mothers: 19.3 %, or 560,000 annual births from adolescent mothers [26].

### Key messages

- The number of women who comply with the recommendation of the WHO to breastfeed for 2 years or more is still low.
- A randomized clinical trial has demonstrated benefits of a pro-breastfeeding intervention aimed at adolescent mothers and maternal grandmothers in the first year of life; this study evaluated results obtained with the same sample at 2 years of age.
- The positive impact of the intervention on the prevalence of breastfeeding observed in the first year of life was not maintained at 2 years of age.
